# Prevalence of cryptococcal antigen positivity among HIV infected patient with CD4 cell count less than 100 of Imam Khomeini Hospital, Tehran, Iran

**Published:** 2017-04

**Authors:** Mahboobeh Hajiabdolbaghi, Saeed Kalantari, Mahin Jamshidi-Makiani, Esfandiar Shojaei, Ladan Abbasian, Mehrnaz Rasoulinezhad, Katayoun Tayeri

**Affiliations:** 1 Iranian Research Center for HIV/AIDS, Iranian Institute for Reduction of High-Risk Behaviors, Tehran University of Medical Sciences, Tehran, Iran; 2 Antimicrobial Resistance Research Center, Iran University of Medical Sciences, Tehran, Iran

**Keywords:** Cryptococal antigen, HIV, Lateral flow assay

## Abstract

**Background and Objectives:**

Cryptococcal meningitis is one of the main opportunistic infections associated with human immunodeficiency virus (HIV) infection. Despite the present and increasingly availability of specific treatment for cryptococcosis, the mortality rate of this infection is still high, particularly in patients with advanced immunsupression and advanced cryptococcal diseases.

**Materials and Methods:**

This Prospective Cohort study was conducted at Imam Khomeini hospital in Tehran, Iran. Serum cryptococcal antigen was detected using the Lateral Flow Assay (LFA) There were 86 HIV-infected patients included in this study.

**Results:**

There were 86 HIV-infected patients in this study. The prevalence of positive serum cryptococcal antigen was 0% (0 of 86).

**Conclusion:**

The prevalence of cryptococcal infection among patients with advanced acquired immunodeficiency syndrome (AIDS) in the Iran is very low (<3%) thus the screening test for cryptococcal antigenemia dose not save lives and is not cost-effective in Iranian population.

## INTRODUCTION

Cryptococcal infection is the main common opportunistic fungal infection in HIV-positive patients. It is a leading cause of mortality in HIV-positive patients in developing countries. Cryptococcal meningitis is one of the main opportunistic infections associated with human immunodeficiency virus (HIV) infection (1). The worldwide burden of Cryptococcal meningitis among patient with HIV/ AIDS was estimated in 2009 to be 957, 900 cases, and approximately 624, 700 deaths annually (2).

Despite the present and availability of specific treatment for cryptococcal infection, the mortality rate is still high, particularly in patients with advanced immunsupression and advanced cryptococcal diseases. Prevention of cryptococcal infection by routine screening with serum cryptococcal antigen for diagnosis of early cryptococcosis and immediate treatment could be a practical approach for this infection (3).

The three methods for diagnosis of cryptococcal infection have been the India ink stain of body fluids for encapsulated yeasts, culture of body fluids, and immunoassay for CrAg. India ink lacks sensitivity and is often negative in patients, and requires experience of laboratory personnel. Culture may also have low sensitivity, can take more days to a result, and require large specimen volumes. CrAg in samples can be determined by three method contain latex agglutination, ELISA and now, by lateral flow immunoassay (4).

The cryptococcal antigen lateral flow assay is an immunochromatographic policy for the qualitative or semiquantitative detection of capsular polysaccharide antigens of complex of *Cryptococcus* species (*Cryptococcus neoformans* and *Cryptococcus gattii*) in serum and cerebrospinal fluid (CSF). The CrAg lateral flow assay is a prescription use laboratory assay, which can use in the diagnosis of cryptococcosis.

Recent studies have reported that screening of serum cryptococcal antigen may be useful in AIDS patients with CD4 cell count less than 100cells/mm^3^ in regions with highly endemic for cryptococcal infection (5). There are few data from Iran on the prevalence of cryptococcal antigenemia.

The purposes of this study were to determine the prevalence of serum cryptococcal antigen in HIV-infected patients with CD4 cell counts less than 100, to determine the risk factors for positive serum cryptococcal antigen, and to assess the incidence of cryptococcal disease during 6 mounth follow-up period after negative cryptococcal antigen screening.

## MATERIALS AND METHODS

This Prospective Cohort study was conducted at Imam Khomeini hospital in Tehran, Iran (a referral hospital with patients from all regions of the country). The study was carried out between January and septamber 2014 and followed the patient for 6 mounth after primary test with clinical findings.

Inclusion criteria were all HIV- infected patients who had CD4 count less than 100 and had been screened with serum cryptococcal antigen. Exclusion criteria included having primary prophylaxis for cryptococcosis or having past history of or current cryptococcal infection.

The data including demographic information, history of AIDS-defining illnesses, clinical symptoms and signs, laboratory data, treatment and outcomes were collected.

Serum cryptococcal antigen was detected by using the Lateral Flow Assay (LFA) test. The CrAg Lateral Flow Assay (LFA) is a dipstick sandwich immunochromatographic assay. For the qualitative procedure, specimens are diluted 1:2 in Specimen Diluent and analyzed (5).

## RESULTS

There were 86 HIV-infected patients included in this study. The mean age was 35.6 (9–56) years and 74.4% were male. 17.4% (15 of 86) had opportunistic infection at time of collecting of serum sample. The mean CD4 count was 45 (5–100).

The prevalence of positive serum cryptococcal antigen was 0% (0 of 86).

None of the patients (0%) with negative serum cryptococcal antigen had developed cryptococcal meningitis after 6 mounth follow up.

## DISCUSSION

Results from this study indicated a 0% prevalence of cryptococcal antigenemia in advanced AIDS patients. Existing prevalence data for CrAg antigenemia in HIV positive patients range from as low as 2% in northern Vietnam to 21% in Benin City, Nigeria (6).

Early diagnosis and treatment is important to reducing cryptococcal meningitis and mortality of this infection. Cryptococcal meningitis is a subacute meningitis that the polysaccharide CrAg is detectable in serum of patient a median of 3 weeks prior to begining of clinical symptoms. The subacute nature of infection allows for effective interventions. Methods to prevent the cryptococcal meningitis and related mortality would include: 1) earlier HIV positive diagnosis and ART initiation prior to progressive to AIDS, 2) primary prophylaxis with antifungal agent (fluconazole) in persons with AIDS, and 3) screening and treatment for occult cryptococcal infection. Both earlier ART and primary fluconazole prophylaxis are effective interventions for prevention (7).

The 2009 US Department of Health and Human Services guidelines for managing opportunistic infections believe that because the low incidence of cryptococcal disease, routine testing for asymptomatic patient for serum cryptococcal antigen is not recommended and also primary prophylaxis is not recommended in the United States (8). But in recent study the prevalence of cryptococcosis in patients with AIDS in the United States was 2.9% that is high, prevalence that screening for cryptococcal antigenemia might be cost-effective (9).

One study showed that 3% prevalence of cryptococcal antigenemia is the cutoff that in the lower of this prevalence cost of treating Cryptococcal meningitis with amphotericin B is greater than the cost of screening. This means that cryptococcal screening is cost-effective in populations that the prevalence of antigenemia is greater than 3% (7). The WHO Rapid Advice Guideline Development Group recommended routine screening of plasma or serum for CrAg in ART-naïve adults. CrAg-positive patients should be given preemptive fluconazol therapy to reduce the progressive to cryptococcal disease. Such screening is recommended in HIV positive patient with CD4 counts less than 100 cells/mm3 and where this patients has a high prevalence of cryptococcal antigenemia (≥3%) (7).

## CONCLUSION

Serum cryptococcal antigen screening has an important role for the early diagnosis and treatment of cryptococcal infection in HIV-infected patients with low CD4 cell counts, particularly in patients with CD4 cellcount of <100 cells/mm ^3^ . This study showed the prevalence of cryptococcal infection among patients with advanced acquired immunodeficiency syndrome (AIDS) in the Iran is very low (<3%) thus the screening test for cryptococcal antigenemia dose not save lives and is not cost-effective in Iranian population.
